# Challenges in Estimating Insecticide Selection Pressures from Mosquito Field Data

**DOI:** 10.1371/journal.pntd.0001387

**Published:** 2011-11-01

**Authors:** Susana Barbosa, William C. Black, Ian Hastings

**Affiliations:** 1 Molecular and Biochemical Parasitology Group, Liverpool School of Tropical Medicine, Liverpool, United Kingdom; 2 Department of Microbiology, Colorado State University, Fort Collins, Colorado, United States of America; Johns Hopkins Bloomberg School of Public Health, United States of America

## Abstract

Insecticide resistance has the potential to compromise the enormous effort put into the control of dengue and malaria vector populations. It is therefore important to quantify the amount of selection acting on resistance alleles, their contributions to fitness in heterozygotes (dominance) and their initial frequencies, as a means to predict the rate of spread of resistance in natural populations. We investigate practical problems of obtaining such estimates, with particular emphasis on Mexican populations of the dengue vector *Aedes aegypti*. Selection and dominance coefficients can be estimated by fitting genetic models to field data using maximum likelihood (ML) methodology. This methodology, although widely used, makes many assumptions so we investigated how well such models perform when data are sparse or when spatial and temporal heterogeneity occur. As expected, ML methodologies reliably estimated selection and dominance coefficients under idealised conditions but it was difficult to recover the true values when datasets were sparse during the time that resistance alleles increased in frequency, or when spatial and temporal heterogeneity occurred. We analysed published data on pyrethroid resistance in Mexico that consists of the frequency of a Ile1,016 mutation. The estimates for selection coefficient and initial allele frequency on the field dataset were in the expected range, dominance coefficient points to incomplete dominance as observed in the laboratory, although these estimates are accompanied by strong caveats about possible impact of spatial and temporal heterogeneity in selection.

## Author Summary

The emergence and spread of insecticide resistance compromise the control of mosquito borne diseases such as dengue or malaria, which are responsible for millions of deaths every year in tropical and subtropical areas. There are currently no easily implemented methodologies to quantify the strength of selection for resistance occurring in nature. Using field data from Mexico on the frequency of an allele mutation conferring resistance in the mosquito *Aedes aegypti* we use maximum likelihood (ML) to estimate the selection and dominance coefficients driving the evolution of resistance. We explored the impact of poor data collection, data that combine information from different locations and the consequences of selection and dominance coefficients varying over the sampling time period. The ML method can accurately estimate these parameters with simulated data in ideal sampling situations but it is difficult to recover true values when spatial and temporal heterogeneity occurs. The analysis highlighted factors relevant to field work such as the need for frequent surveillance in discrete sentinel sites.

## Introduction

Mosquito-borne diseases are prevalent in the tropics and subtropics and constitute a large proportion of the health problems in developing countries. The major mosquito vectors occur in the genera *Culex*, *Aedes*, and *Anopheles*, which transmit *Filaria spp*, Japanese encephalitis, dengue and yellow fever viruses and malaria. The control of vector populations is often based on insecticides, such as larviciding, indoor residual spraying (IRS) and personal protection through insecticide treated materials (ITM) and their use has been shown to have a powerful impact on mosquito abundance and disease transmission [1]. The foraging and resting behaviors of mosquitoes ensure a number of potentially lethal interactions with insecticide-treated surfaces during parts of the mosquito lifecycle [2], but prolonged exposure to an insecticide over many generations runs the risk that mosquitoes will develop resistance. In general only four different chemical classes of synthetic insecticides are used in the field: organochlorines, organophosphates, carbamates and pyrethroids. Pyrethroids are particularly important because they are the only class of insecticides recommended by The World Health Organization to use on ITM. Since ITM are being widely distributed across malaria and dengue affected countries and pyrethroids are employed in some areas for agricultural pest control, there is concern that the emergence of resistance will compromise these efforts. The widespread use of a small portfolio of compounds against large mosquitoes populations with many generations per year (estimated at 12 per year for *Anopheles gambiae* [3] and 20 for *Aedes aegypti* [4]) have raised fears that high levels of resistance may arise very quickly.

**Figure 1:**
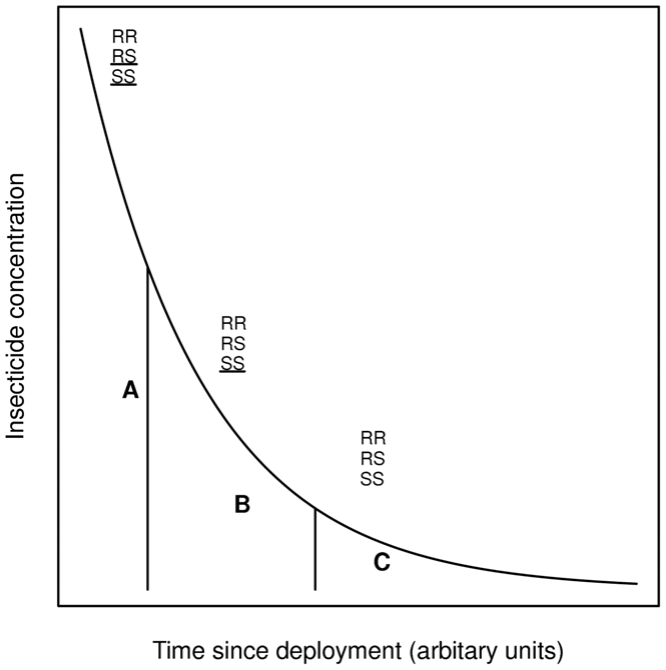
The typical change in insecticide concentration in the field over time. As concentration decays with time after deployment there is a differential survival of genotypes. In period A the RR genotype will survive while the RS and SS dies: this makes the R allele recessive in this period. In period B both RR and RS survive making the R allele dominant in this period. In period C all genotypes can survive so no selection occurs. These are windows of selection, adapted from [37].

We consider the problem of measuring the strength of selection for insecticide resistance in mosquito field populations and show how changes in the frequencies of the alleles at a single locus can be used to estimate the selection acting on each genotype. This type of data is collected for the identification of genetic mechanisms of resistance and/or during monitoring programs of vector control campaigns. The method we developed extends that described earlier by DuMouchel and Anderson in 1968 [5] for laboratory populations. Laboratory based conditions differ significantly from the field. In the laboratory insecticide assays are conducted over standardized range of doses and concentrations that do not account for field situations such as decay rates and exposure characteristics. Following insecticide deployment in the field, concentration decreases and there is a selective window of time at lower concentrations (see Figure 1), where resistant heterozygotes do not die but susceptible homozygotes are still killed, therefore acting as dominant when under more standardized conditions it may appear to be recessive. This is relevant because dominance relationships between susceptible and resistance alleles affect the rate of spread of resistance.

Using a maximum likelihood (ML) procedure and a recursive genetic model that tracks the changes in the resistance allele frequencies at a single locus it is possible to estimate a selection coefficient (s), a coefficient quantifying dominance (h) and the initial frequency of the resistance allele (*p_0_*_,_), key parameters that determine the dynamics of resistance. The model provides a straightforward way to obtain these values with the least complex dataset possible. However, field data on the spread of resistance is often suboptimal: datasets may be small, may only track one period of dynamics (typically early and late stage of spread) or may be pooled from different locations. In this paper we discuss the challenges associated with this approach. We used published data on pyrethroid resistance from *Aedes aegypti*, throughout Mexico [6] on the frequency of the Ile1,016 mutation, one of the mutations in the voltage-gated sodium channel gene known to confer resistance to pyrethroids (this is known as knockdown resistance, a term applied to insects that fail to lose coordinated activity immediately after exposure).

## Model and Methods

The genetic model we employ assumes a single autosomal locus conferring insecticide resistance in a diploid sexually reproducing population, with non-overlapping generations and assuming random mating; these are standard assumptions in population genetics models. There are two possible alleles, resistant (R) or susceptible (S), and three possible genotypes (SR, RR, SS). The fitness coefficient, which is a measure of survival and reproduction of the different genotypes, was defined as 1 for the susceptible homozygotes SS, 1+*s* (*s* is the selection coefficient) for resistant homozygotes RR and 1+*hs* (*h* is the dominance coefficient) for heterozygotes SR. The level of dominance is a measure of the relative position of the phenotype of the heterozygote relative to the phenotype of the two corresponding homozygotes. Complete dominance for susceptible allele is represented by *h* = 0 and complete dominance for resistance allele by *h* = 1, alleles are codominant or additive when *h* = 0.5. The fitness coefficients are composite measures of fitness in both the exposed and unexposed mosquitoes groups and are assumed to be the same for males and females.

We also assume a large population, so that genetic drift can be ignored, which enabled us to predict the frequency of the resistant allele at any time *t* according to the recursion expression:
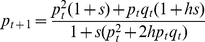
(1)Where:

*p_t_*: frequency of the resistant allele at time/generation *t*
*q_t_* = 1−*p_t_*: frequency of the susceptible allele at time/generation *t*

This recursive equation is the basic formula of selection of a favourable gene [7], [8]. We defined the allele initial frequency *p_0_*, as the frequency in the first sampling time point, set as generation 0. Each subsequent generation can be converted onto a real timescale of years by assuming a constant number of generations per calendar year.

The ML approach to estimate the unknown parameters *h* and *s* and *p_0_*, based on this genetic model, involved selecting initial values of *s*, *h*, and *p_0_*, and then testing how well the predicted allele frequencies matched those observed in the dataset.

Field datasets usually consist of the number of resistant alleles *x_t_* and the total number of sampled alleles *n* at different time points *t*. The probability of observing *x* resistant alleles among *n* alleles follows a binomial distribution, with a probability of success (being a resistance allele) *p* for each sampled time point *t*.
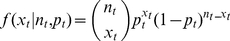
(2)Where:

*p_t_*: probability of sampling an R allele, i.e, probability of a success

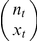
: combinatorial term to account for the number of ways of sampling *x* resistant alleles among *n* total alleles

The corresponding binomial likelihood function is:
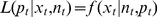
(3)The likelihood function returns the likelihood of the value *p_t_* given the observed data of *x_t_* resistant alleles among the sample of *n_t_* at each generation. Essentially, it tells us how consistent the data are with predicted values of *p_t_* (Equation 1). The likelihood value for the dataset is the product of the likelihoods across the entire sample:
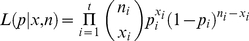
(4)We implemented this ML methodology in *R* [9] using *constrOptim()* function (from *stats* package) for which it is not necessary to provide analytic derivatives and that can minimize/maximize a function subject to linear inequality constraints. Three constraints on the parameter values were enforced: 0<*p*<1, 0<*s*<1, 0<*h*<1, except when analysing the field data when a constraint on *h* (0–1.5) was imposed. The Nelder-Mead optimization method algorithm was used, that generates a new test position by extrapolating the behavior of the objective function measured at each test point arranged as a simplex. The algorithm then chooses to replace one of these test points with the new test point and the algorithm progresses. The simplest step is to replace the worst point with a point reflected through the centroid of the remaining points. If this point is better than the best current point, then it will expand exponentially along this line. On the other hand, if this new point is not much better than the previous value the simplex returns the previous point. The standard error (s.e) of the estimates was determined by inverting the Hessian matrix evaluated at the ML estimate and the 95% confidence interval endpoints were calculated as *Parameter estimate*±1.96*s.*e*.

Maximum likelihood estimation is an optimization technique and there is no guarantee that the set of parameters that uniquely maximizes the likelihood will always be found because the algorithm may converge onto local optima whose likelihood is below the global maximum. To overcome this problem 1000 runs of the ML iteration procedure were performed in every estimation, with random starting values of the parameter estimates used to initialise the optimization routine [10]. In the analyses described here, the runs that converged to other estimates had ML values sufficiently less than the global maximum that a likelihood ratio test considered them different, so that the set of parameters could be safely discarded. However a small percentage of the runs converged to a set of different parameters with a similar likelihood value that could not be considered different using a likelihood ratio test. The criteria used to exclude these results as potential best estimates was that the estimated value of *h* lay on the boundary of the constrained parameter range and is expected to reflect erratic behavior of the algorithm when using a small sample.

We tested the algorithm and program by analyzing 100 datasets simulated under idealized conditions using Equations 1 and 2. Initial frequency, dominance and selection coefficient were in the ranges 0.01–0.04, 0.3–0.8 and 0.1 to 0.3 respectively, all distributions were uniform. Three parameters values were selected for each dataset and held constant during the simulation, i.e., there was no temporal or spatial variation in parameter values and populations sizes were sufficiently large that stochastic changes in alleles frequencies could be ignored. Data were available for each generation, 100 alleles (50 mosquitoes) were sampled each generation (Equation 2) and the simulations were run until the resistance allele frequency exceeded 0.99. Accuracy of analysis was gauged by the correlation coefficient between true and estimated parameter values, and by checking how frequently the true values fell with the estimated 95% confidence intervals.

Next we examined the impact of suboptimal datasets. Equation 1 was used to predict allele frequency for 120 generations and we assumed that 100 alleles were sampled in each generation. Two optimal datasets with different dominance values were produced to check if the ML method accurately recovered the parameters when data from all generations was available (as above) and to investigate the effect of different degrees of dominance on estimations. Subsets of the data were used to examine the influence of incomplete sampling when only a few generations of data are available, or when only the initial stages of spread are available for analysis.

Field data, collected and analyzed by Garcia *et al.* [6], was available for analysis. There were a total of 78 field collections containing 3,808 *Aedes aegypti* (some as much as 2000 km apart). Each mosquito was genotyped at the Ile1,016 locus. We pooled data from the different locations and analysed it assuming different number of generations of mosquitoes per year (6,9,12,16 and 20), to check the consistency of the estimations. Intuitively, we would expect spatial and temporal variation in the selection parameter in the Garcia *et al.* dataset and in many other datasets obtained under field conditions. It was therefore vital to ascertain how heterogeneity would affect the algorithm's ability to recover the underlying parameters from pooled data. Spatial heterogeneity was investigated by simulating allele frequencies for 80 different locations over 50 generations using Equation 1; 100 alleles were sampled from each generation (Equation 2) and data from each generation in each location were used in the analyses. Parameters p_0_ and *h* were randomly selected from a uniform probability distribution (p_0_∼∪(0,1), *h*∼∪(0,1)) while *s* was randomly drawn from a normal distribution (*s*∼*N*(0.15,0.025)), the constraints on *s* coefficient are within a reasonable range for a field setting. Once selected for a location, the values of *h* and *s* did not change, i.e., there was no temporal heterogeneity. Two simulation strategies were used: (i) p_0_ and *h* were allowed to vary while *s* was held constant at 0.1, 0.3, 0.6, 0.8 or 1, (ii) *p_0_*, and *s* was allowed to vary while *h* was held constant at 0, 0.25, 0.5. 0.75 or 1. The data across the simulated locations were pooled for analyses. Each simulation strategy was run 300 times giving a total of 300×5 = 1500 per strategy. The mean values of each parameter over all simulated locations was assumed to be the true value and the accuracy of the program was, as before, gauged by the correlation coefficient between the estimated and true values, and by the proportion of the true values falling within the 95% CI.

The effect of temporal heterogeneity in estimations was also investigated by varying *s* and *h* over 50 generations in a single location, i.e., different *s* and *h* values in different generations. The distribution of values were the same as those used for spatial heterogeneity. Three scenarios were considered: (i) *s* and *h* both varied over generations, (ii) *h* could vary while *s* was held constant, (iii) *h* could vary while *s* was held constant. In the simulations of spatial heterogeneity the values of *h* and *s* had to be fixed across locations (e.g. *h* = 0, 0.25, 0.5. 0.75 or 1) but in the simulation of temporal heterogeneity only one location was examined in each simulation so the values could be drawn from the underlying distributions. As before, 300 datasets were produced for each scenario but because the fixed values of *h* and *s* could be drawn from a distribution, the total number of runs was 300×3 = 900. As before, the performance of the algorithm under conditions of temporal heterogeneity was assessed by defining the true value as mean over the generations, and calculating the correlation coefficient between the estimated and true values and how frequently the true value was included in the 95% confidence interval.

Finally, it is important to note two features of our analyses that may not be obvious to non-specialists. Firstly, that the genetic parameters *h* and *s* describe the overall, net rate of spread of resistance alleles through natural populations and cannot formally distinguish where selection is acting. For example, they cannot determine whether selection was acting differentially on the adult or larval stages, whether fitness costs were associated with resistance, whether there was differential selection on the sexes, nor whether killing was likely to be in early or later adult stages, the latter being a topic of contemporary interest given recent suggestion by Koella and colleagues [11] that killing older adults will reduce the selective pressures for insecticide resistance. Secondly, the analyses were designed to recover the genetic parameters that resulted from past control program and, as such, they cannot explore the issue of how differing patterns of insecticide deployment drive resistance. This require a separate, formal modelling approach explicitly designed to investigate the differing impact of deployment strategies on driving resistance. These analyses have been described elsewhere, particularly for the agriculture pesticides [12]–[15].

## Results

The analysis of idealized datasets (Table 1) suggest ML can accurately recover the underlying parameter values from optimal simulated data.

**Table 1:** Details of 100 idealized simulated datasets.

	*p_0_*	h	s
Parameter range	0.01–0.04	0.2–0.8	0.1–0.3
r *	0.94	0.99	0.99
TV (%)*	91	92	97
[ ]*	0.021	0.014	0.002

The simulated datasets were used to check the precision and accuracy of the ML procedure.

*r correlation coefficient between original value and estimate, TV percentage of true values in the estimates 95% confidence interval and [ ] mean range value of the confidence interval.

**Figure 2:**
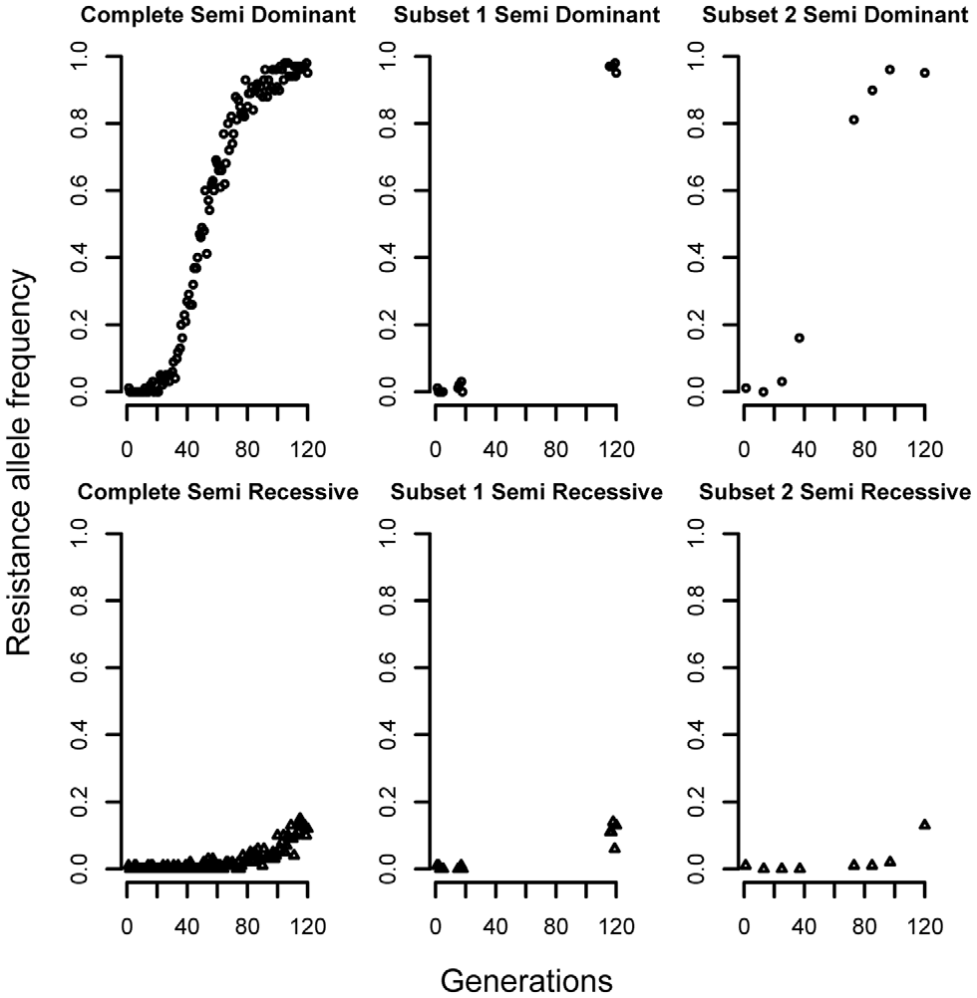
Simulated evolution of resistance allele frequency over 120 generations under two different scenarios of dominance relationship and analysing the full dataset or subsets of data. Specifications in Table 2.

Figure 2 shows six example simulations of the increase in resistance allele frequencies over 120 generations, under two dominance conditions (semi-recessive, *h* = 0.2 bottom panel, and semi-dominant, *h* = 0.8 top panel). Values of *p_0_*, *s*, and *h* appear in Table 2. The program appears less accurate when analysing subsets of the original data, particularly if the resistance allele is semi-recessive. When the resistance allele was semi-dominant, resistance increased rapidly and the true estimates were recovered if the subset included points that captured the pattern of increase, such as the subset 1. When the resistance allele was semi-recessive, the frequency was maintained at low levels for a long period, the true parameters values were either recovered (Subset 1) but within confidence intervals that were so large as to be uninformative, or were not even contained in the confidence intervals (Subset 2) even with the inclusion of the last generation, the only sampling point in the subset that captures the incipient frequency rise. The ML parameter estimates in Table 2 were achieved in 34 to 83% of the 1000 ML runs indicating that a significant proportion of the estimation routines converged onto local maxima.

**Table 2:** Specifications of datasets of Figure 2.

		True			Estimates [95% CI]		
Dataset	Generations	p_0_	h	s	p_0_	h	s
Complete	1∶120	0.001	0.2	0.2	0.0010 [0, 0.0018]	0.17 [0.13, 0.38]	0.24 [0, 0.48]
		0.001	0.8	0.2	0.0009 [0.0005, 0.0013]	0.81 [0.77, 0.84]	0.20 [0.19, 0.21]
Subset 1	1∶5,15∶18,116∶120	0.001	0.2	0.2	0.0025 [0, 0.0062]	0.45 [0, 1.5]	0.07 [0, 1]
		0.001	0.8	0.2	0.0016 [−0.0017, 0.0049]	0.77 [0.36, 1.18]	0.19 [0.08, 0.29]
Subset 2	1,13,25,37,73,85,97,120	0.001	0.2	0.2	0.0014 [0, 0.0030]	0.02 [0, 0.18]	1.00 [0.96, 1]
		0.001	0.8	0.2	0.0009 [−0.0007, 0.0025]	0.80 [0.64, 0.96]	0.20 [0.16, 0.25]

Sampled generations and true parameter values and ML parameter estimates with respective 95% confidence intervals.

Analysis of the *Aedes aegypti* dataset resulted in the parameter estimates in Table 3. These ML estimations were obtained assuming 6, 9, 12, 16 and 20 generations per year. The estimation converged on the same ML value 76 to 96% of the runs. With a small percentage of the runs (0.005 to 0.16%) converged to a set of different parameters with a similar likelihood value but which were excluded because the estimated value of *h* was on the boundary of the constrained parameter range. The estimates of *p_0_* and *h* were highly consistent irrespective of assumed number of generations per year and ranged from 0.0032 to 0.0035 and from 0.77 to 0.78, respectively. As expected the *s* was strongly dependent on the assumed number of generations per year and ranged from 0.042 to 0.15.

**Table 3:** Estimated *p_0_*, *h* and *s* parameters from field data.

Parameter	Generations/year	Best value	95% Confidence interval	
*p_0_*	6	0.0032	0.0032	0.0032
	9	0.0033	0.0033	0.0033
	12	0.0034	0.0034	0.0034
	16	0.0034	0.0034	0.0034
	20	0.0035	0.0035	0.0035
*h*	6	0.77	0.76	0.78
	9	0.77	0.76	0.78
	12	0.77	0.76	0.78
	16	0.78	0.77	0.78
	20	0.78	0.77	0.78
*s*	6	0.15	0.14	0.16
	9	0.096	0.090	0.101
	12	0.071	0.060	0.081
	16	0.053	0.048	0.057
	20	0.042	0.038	0.046

The dataset corresponds to field collected data on Ile1,016 resistance allele frequencies in *Ae. Aegypti* from Mexico. Assuming 6, 9, 12, 16 and 20 generations per year.

**Figure 3:**
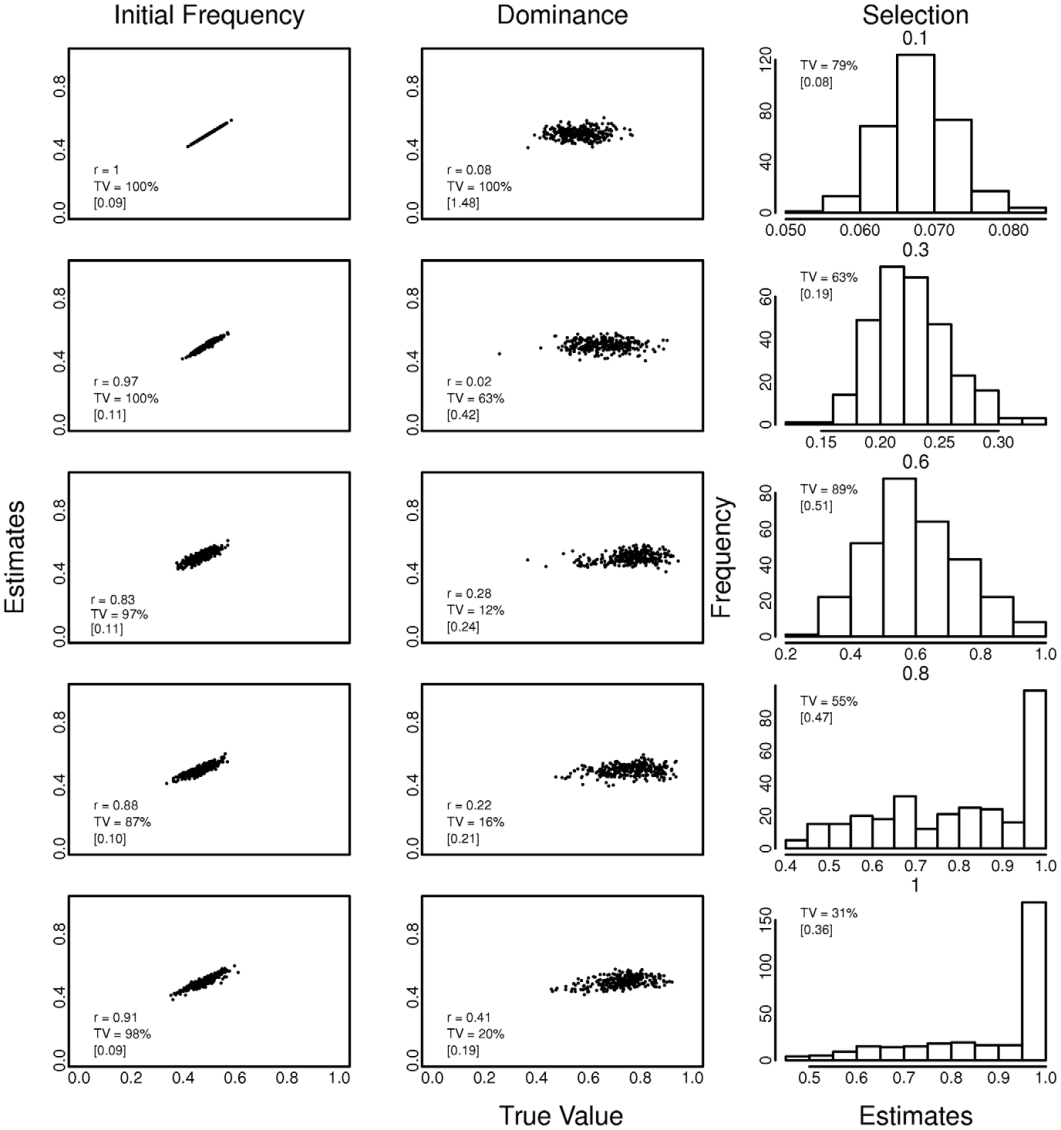
Effect of spatial heterogeneity (pooled data from 80 simulated locations) on estimates of initial allele frequency, dominance and selection parameters. The value of the selection coefficient was held constant at 0.1, 0.3, 0.6, 0.8 or 1 in all locations and in every generation, hence there are five rows of results corresponding to each of the 5 values of the selection coefficient. Dominance (*h*∼∪(0,1)) varied between simulated locations, but was constant over time within each location. The true value is the mean parameter value over all locations. The Pearson correlation coefficient (r) is between estimated and true values. TV is the percentage of the true values that are included in the 95% confidence interval of the estimate. [ ] is the mean width of the 95% confidence interval in all runs.

**Figure 4:**
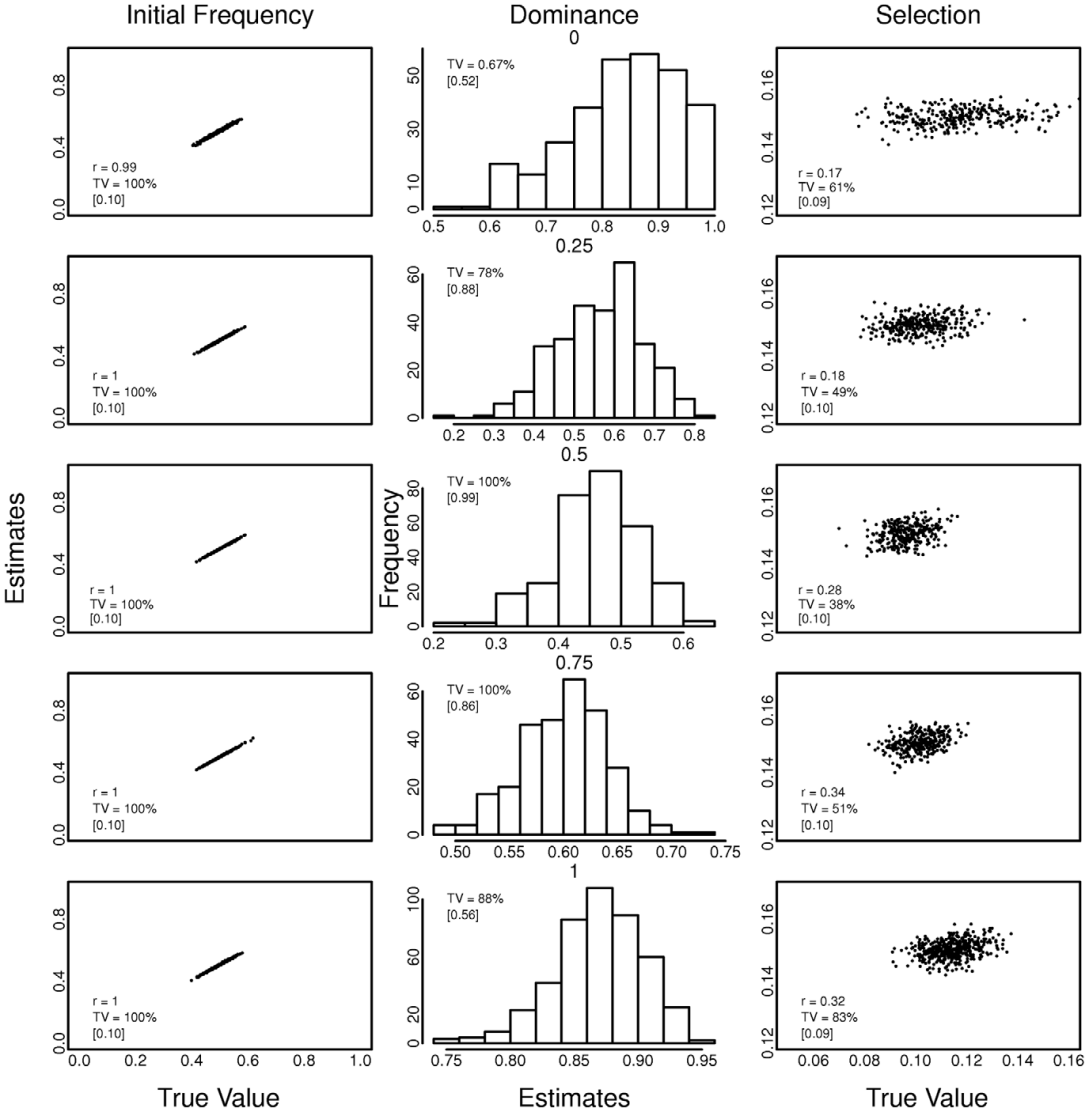
Effect of spatial heterogeneity on estimates of initial allele frequency, dominance and selection parameters. The value of the dominance coefficient was held constant at 0, 0.25, 0.5, 0.75 or 1 in all locations and in every generation, hence there are five rows of results corresponding to each of the 5 values of the dominance coefficient. The value of the selection coefficient (*s*∼*N*(0.15, 0.025)) varied between locations, but was held constant over time in each location. The true value is the mean parameter value over all locations. The Pearson correlation coefficient (r) is between estimated and true values. TV is the percentage of the true values that are included in the 95% confidence interval of the estimate. [ ] is the mean width of the 95% confidence interval in all runs.

Results from spatially heterogeneous datasets pooling data from 80 different locations are shown in Figures 3 and 4. The algorithm appears unable to consistently obtain accurate estimations of the parameters *s* and *h* under such heterogeneous settings, manifested by low values of correlation coefficients and many true values outside the 95% confidence interval of the estimate. For example, with the dominance estimations in Figure 3 when selection was constant at 0.6, only 12% of the true values fell within the confidence interval. Initial frequency values were accurately recovered in all simulated scenarios, possibly due to the the recursion dependency on the initial frequency. However, the estimation of selection and dominance coefficients was achieved with very low values of correlation coefficients between the estimates and the mean of the parameter over the 80 simulated locations (not very precise), in all different hypothetical scenarios.

Additionally, if most of the values were in the confidence interval, the mean range of the interval was as wide as the parameter range. For example in Figure 4 note that when dominance is constant at 0.75, 100% of the true values are in the confidence interval, but the average mean range is 0.86 (the parameter range is 0–1). The plotted simulated data and estimates do not traverse the entire range of the parameters values because they are the mean over the 80 locations, the central limit theorem predicts that these estimates will converge to the center of the distribution.

**Figure 5:**
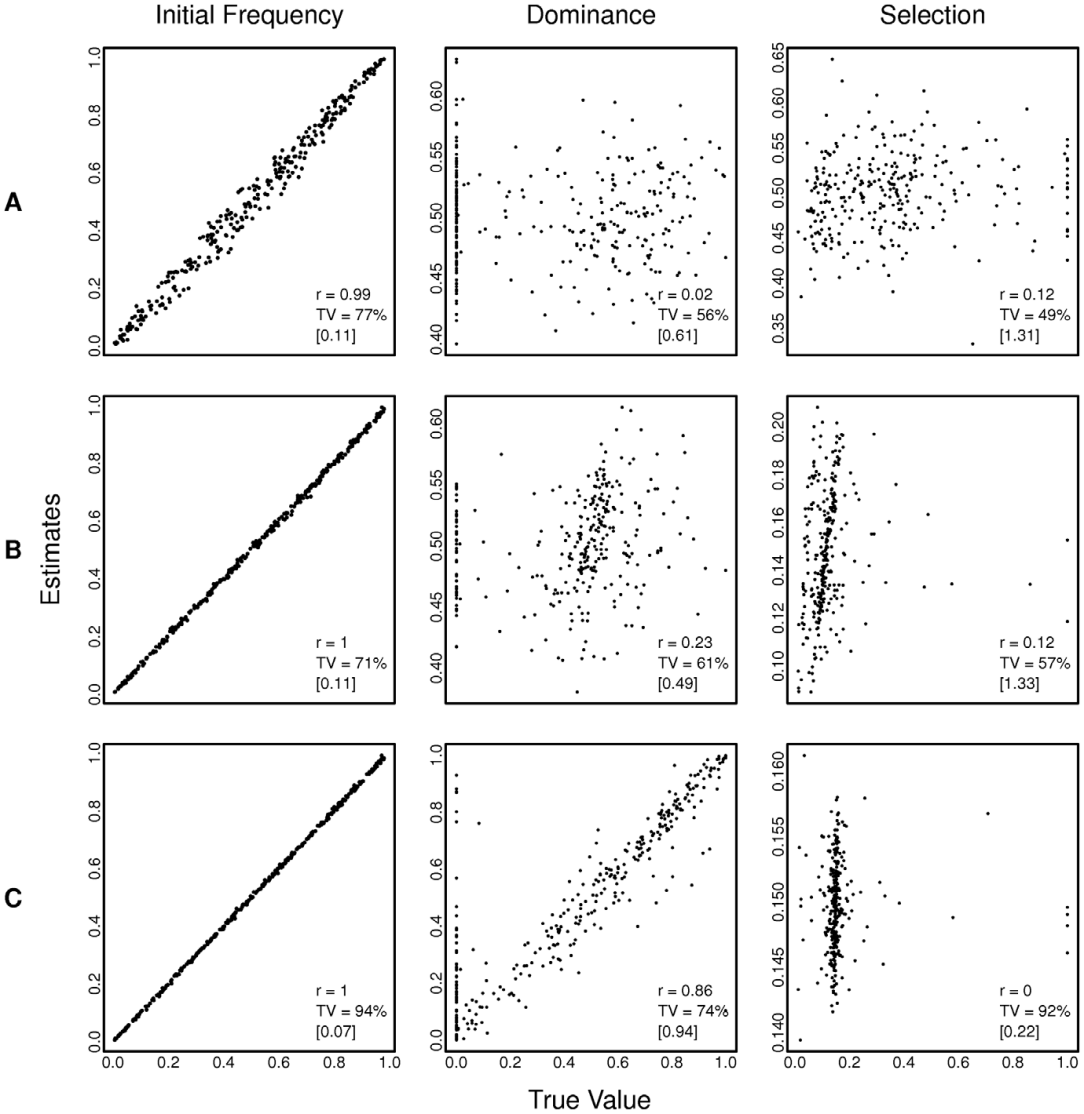
Effect of temporal heterogeneity on estimates of initial allele frequency, dominance and selection coefficients parameters. Three different scenarios were simulated: (A) dominance and selection are different in every generation, (B) selection coefficient was held constant in all generations but dominance was allowed to vary, (C) dominance was held constant in all generations while the selection coefficient was allowed to vary. The Pearson correlation coefficient (r) between estimate and true value is shown. TV refers to the percentage of the true values that are included in the 95% confidence interval of the estimate. [ ] is the mean range of the 95% confidence interval in all runs.

Simulations of a location with temporal heterogeneous selection pressure (dominance and/or selection changing in every generation) are shown on Figure 5. Again, the model does not accurately recover the true parameters under conditions of temporal heterogeneity. The exception was the dominance parameter when it was held constant in a particular location with selection varying in each generation, the correlation coefficient between the estimate and the mean dominance value over the 80 locations was 0.86.

## Discussion

Insecticide resistance research is largely focused on the identification of the mechanisms responsible for resistance, and whether the genetic mechanism is monogenic or polygenic, general or population specific and if there are associated fitness costs and developmental patterns [16]. The emergence and spread of resistance is well documented, but there is still a worrying lack of quantification of the evolution dynamics in populations under control [17] and its persistence in populations following cessation of control. The quantification of the strength of selection acting in the wild has previously been attempted using direct laboratory and field trials, and indirect approaches using a variety of data, including patterns of DNA variability and spatial and temporal changes in allele frequencies [17], [18]. Selection acting on insecticide resistance genes in the field was first estimated using genetic models for species in the genera *Anopheles* by Curtis *et al.* [19] and Wood and Cook [20], both were based on the observed changes in gene frequency over regular intervals and the latter also discussed estimation by deviations from the expected Hardy-Weinberg equilibrium frequencies. Both methods assumed a fixed level of effective dominance under field conditions. A recent example is the estimation of relative fitness by Livingston and Fackler [21] for pyrethroid resistance in insects that infest crops. In this case the magnitude of the estimates were similar to those obtained using traditional laboratorial direct approaches using non linear least squares estimation. The most refined work that we are aware of, quantifies selection coefficients and costs associated with resistance for *Culex pipiens* in Southern France, using spatial information from clines to estimate selective advantages and costs, and temporal information from a long-term survey to estimate the selection coefficients of alleles in each environment using a standard ML estimation approach [22], [23].

We have described a ML method for simultaneously estimating the selection and dominance coefficients and an initial resistance allele frequency similar to that of [5], but we also tackled the effects of spatial and temporal differences in selection intensity that can arise as a result of different strategies of deployment of the insecticide, migration patterns and/or infrequent and sparse field sampling of mosquitoes. The approach described in this paper was accurate with simulated data but proved less robust when analysing few intermediate allele frequencies, especially when the resistance allele is recessive. The reason is that all resistance dynamics start from the same point (very low frequency) and end at the same point (very high frequencies) but in the absence of intermediate time points it is impossible to reconstruct the dynamics in between. If the sampling period covers only the onset of resistance or the final stages, when resistance is close to fixation, the accurate estimation of selection and dominance coefficients can be difficult. The estimation is problematic because in the early stages heterozygotes prevail in the population, with a fitness W_rs_ = 1+*hs* for which there are a range of values of *h* and *s* that yield the same product *hs*. This is illustrated using subsets in Figure 2. The true values of *s* and *h* were 0.2 and 0.2 but the estimates were 0.45 and 0.07 (Table 2). The situation was even worse for subset 2 of the data (Figure 2) where the analysis inferred a completely different trajectory of resistance spread and the true values of *h* = *s* = 0.2 were estimated as *h* = 0.02 and s = 1.0 (Table 2). Once again, note that the fitness of the heterozygote was estimated as 1+*hs* = 1.02 which was relatively close to the true value of 1.04 and that it is the predicted value of the homozygotes, which were largely absent from this subset of the data, that were badly estimated (as W_rr_ = 2.0 rather than the true value of 1.2). Nevertheless, the calculated fitness (*1+hs*) is very similar (1.04 and 1.03). On the other extreme, when resistance is almost fixed, there will be mainly homozygotes in the dataset (with fitness W_rr_ = 1+*s*), so estimating a dominance value will also be problematic because of the lack of heterozygotes (with fitness W_rs_ = 1+*hs*). Unfortunately, this is a very common type of data where genetic surveys initially indicate resistance was absent then, once its presence was detected, a second survey was run and higher levels were detected. Our analyses indicate that it is highly unlikely that any robust genetic parameters can be obtained from these kind of fragmented datasets. Future surveillance surveys should consequently be optimized by choice of a proper sampling strategy and timeframe. It is therefore of extreme importance to sample as many generations as possible, even if it means collecting fewer individuals. There is an important difference between standard statistics and ML estimation. In standard statistics, the 95% CI should capture the likely variation in magnitude of parameter estimates. In ML it only captures the likely variation provided the model has identified the correct trajectory of allele frequency changes. This is problematic in incomplete datasets where many trajectories may provide similar fits to the observed data. It is absolutely essential to run numerous analyses from randomly selected starting parameter values to check for the presence of numerous trajectories of similar ML but with widely different parameter estimates.

Pooling data from different locations can be seen as a reasonable option to minimize the lack of sampled generations and small sample size. The Ile1,016 mutation frequency dataset of Garcia et al. [6] provided the opportunity to apply the model to real field data. This data contains allele frequencies of mosquitoes collected in 78 different locations around Mexico since 1999. Insecticide use was not uniform across cities and towns in Mexico and will probably differ between years and in addition migration will probably lead to different initial resistance allele frequency. The estimates obtained from simulated pooled data demonstrated that this kind of data-pooling, which is probably inevitable in most surveys, is not very robust. The coefficients reported in Table 3 should simply be recognized as a rough estimate between the years 1999 to 2008 and that they may vary, albeit by an unknown amount, over time and space.

Equation 1 describes a highly idealized population, i.e., one that is large, randomly mating, and homogenous in time and space. It is therefore important to consider the extent to which our population differs from this paradigm and what consequence this may have for the results. A large population is required so that we can ignore genetic drift, i.e., random fluctuations in allele frequency around our predicted values. Drift is important in laboratory studies (see [24] for discussion) but in natural populations there is a consensus that genetic drift can be ignored provided 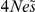
>10 where *Ne* is effective population size [25] and 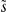 is the weighted mean fitness of the resistance heterozygotes and homozygotes. Estimates of *Ne* provided by [26] for *Aedes Aegypti* ranged from 10–22 in different regions of Mexico. These estimates seem intuitively to be very small. The most likely explanation is that they are measure of historical population size, so may have been caused by founder effects and population bottlenecks in the distant past. Estimates of contemporary population sizes are more appropriate in the current context and most estimates of contemporary effective population sizes of vectors are much higher, for example, in the region of 1000+ for *Anopheles gambiae* [27]–[29]. It would be possible to introduce the effects of drift by simulating small populations sizes and sampling (with replacement) the parents of the next generation. However one of the key conclusions of this study is the difficulty of obtaining good quality estimates of genetic parameters from field data, so we prefer to ignore the effects of drift, and simply point out that the stochastic variation introduced by drift will likely further decrease our ability to recover accurate genetic parameter values from field data.

The second requirement, that mating occurs at random is unlikely to be true given the geographical scale of our surveys. It would be relatively straightforward to incorporate this effect by including Wrights F statistics in Equation 1. However, there was no evidence of significant departure from Hardy-Weinberg in our dataset (results not shown) so this strategy was not required. The assumption that the population is homogenous in space is clearly untrue. Pooling of data from different regions was required to increase sample size and frequency because mosquito collections were not uniform at the same location. The simulation results demonstrate the dangers of this approach and work on malaria vectors in Africa show unpredictably high levels of heterogeneities in resistance even across relatively small distances [30]. As mentioned by [5] simple models cannot account for the alteration of selection pressure by long term changes in the environment. More complex models that consider geographic clines and the antagonist effect of selection-migration, should be more accurate, but the amount of data necessary make the implementation unlikely in most settings. This model in its simplicity presents a straightforward way to obtain estimates of fitness parameters. The fact that only information about resistant allele frequencies is necessary should make it easier to apply, and yet even such a simple data design is difficult to implement.

Nevertheless the estimated value for p_0_ (0.0032–0.0035), was in the higher range of 10^−2^ to 10^−13^ expected when a pesticide is first introduced, based on mutation-selection equilibrium [31]. This initial p_0_ value reflects the frequency prior to the sampling period. Since 1950, vector control programs in Mexico have used a series of insecticides. DDT was used extensively for indoor house spraying from 1950–1960 and was still used in some locations up until 1998. Malathion was later used for ultra-low volume space spraying of wide areas from 1981 to 1999. In 2000, programs switched to permethrin based insecticides [32]. The spread of resistance genes in a treated region will depend on the initial resistant allele frequency and it is known that resistance development in pest organisms can occur within 5–100 generations [31]. The relatively high initial frequency estimated explains the immediate, dramatic increase in frequencies of Ile1,016 from the late 1990s to 2006–2008 [6] neglecting genetic drift.

As expected, the strength of selection increased as the number of generations per year decreased, whereas there was less time to get to the same frequency of resistance allele. Selection coefficients ranging from 0.042 to 0.053 (assuming 20 and 16 generations per year) are similar to the selection coefficients of DDT and dieldrin resistant phenotypes in *Anopheles* mosquitoes that have been previously estimated to be on the order of 0.013–0.061 [16]. The values for 0.071 and 0.097 (12 and 9 generations per year) are in the range of what was estimated for antimalarial drug resistance: 0.05–0.1 [33], however the value of 0.147 for 6 generations was higher than any previous estimates. This is the first time selection for insecticide resistance has been quantified in this species and should be seen as a preliminary estimate.

The estimated values of *h*, 0.77 to 0.78, point to partial dominance of the resistance allele under field settings. Alleles conferring knockdown resistance were found to be to be recessive or semi-recessive in their influence in *Anopheles gambiae s.s.* [34], but there is strong evidence for partial dominance or additive effects of Ile1,016 from two laboratory studies of knockdown and survival in strains or families of *Aedes aegypti* segregating for the Ile1,016 allele. Saavedra-Rodriguez *et al.* [35] found that 127 of 221 heterozygotes recovered from permethrin knockdown and showed later [36] that when considering overall survival the differences among the three phenotypes appear additive. Dominance in the field is dependent on the concentration and decay of the insecticide (see Figure 1), under this situation the resistant allele will be effectively dominant and we think that our results of intermediate dominance of Ile1,016 reflect this effect [37]. This interpretation is supported by Roush and Tabashnik [31], who reported the same situation of partial dominance for cyclodienes and lindane, diazinon, malathion and also for pyrethroids, where 20–60% of the heterozygotes survived exposure in a field setting. There is ongoing debate about differences between laboratory and field settings that extended to the evolution of insecticide resistance itself, some suggesting that resistance in the fields tends to be based on an allele of major effect at a single locus whereas resistance obtained in the laboratory is usually polygenically based [38]. Our results show rapid selection of mutations at a single locus.

The number of generations under natural conditions for this species was estimated at 20 or more among strains in field conditions in Brazil, this leads us to consider the results with the highest number of generations as the most likely, but because *Aedes aegypti* eggs can survive desiccation for months and hatch once submerged in water [39], the number of generations is variable. Nevertheless, the predicted resistance frequency trajectory using equation 1 and the different estimates obtained assuming different generations per year will be approximately the same in a timescale of 20 years.

Most mutations encoding insecticide resistance are expected to incur a fitness penalty, compared to unmutated genes, in the absence of insecticide. There is some field evidence of reduced fitness of Ile1,016 mutations in *Aedes aegypti* in permethrin free environments [6] which leads us to make two technical points. Firstly, that the selection and dominance coefficients reported here are overall values that combine the mutations benefit when encountering insecticide and any fitness effect in insecticide-free areas. Secondly, the method can equally be applied to measure negative selection pressures, (i.e., when a mutation is being lost from a population) from field data on the mutation after insecticide is withdrawn.

Two factors are of particular relevance to field work. Firstly, that surveillance needs to be continuous so that a full dataset covering the whole period of resistance spread becomes available upon which to base these estimates. This may mean monitoring sentinel sites for long periods when resistance is rare or absent, but a continuous dataset is a prerequisite for accurately estimating the dynamics underlying the spread of resistance. Note that a continuous dataset does not necessarily mean collecting samples every generation. The reason the analysis could fail to recover the true parameters (Table 2) was because of large gaps in the survey: simulations of semi-dominant mutations lacked samples from periods of intermediate frequency, while simulations of semi-recessive mutations only contained data from the early stages (Figure 2). Operationally, this suggests that regular, rather than intensive but periodic, sampling is the best strategy. As an example, we re-analysed the semi-dominant dataset but just incorporated samples every 10 generation, i.e., at generations 1, 10, 20, 30:120. This resulted in estimates of *p_0_* = 0.0006 (95% CI: 0.0001–0.0010 ), *h* = 0.89 (95% CI: 0.84–0.95), *s* = 0.20 (95% CI: 0.18–0.21) which are similar to those obtained using data from all generations (Table 2, the dominance coefficient is higher but the confidence intervals overlap). The second point is that dominance levels acting in the field may be much higher than those observed in the laboratory. The most plausible explanation is that mosquitoes in the wild are encountering low levels of insecticide that are insufficient to kill heterozygotes. Increasing dominance greatly increases the rate at which resistance develops. This suggests that insecticide applications should be enforced in such a way that ensure high coverage with high doses. Our results suggest that the doses being applied may be inadequate and that pursuing the current deployment settings will lead to the rapid increase of resistant mosquitoes and eventually to the complete inefficiency of permethrin in the combat of dengue in Mexico.
